# Characterization of a lytic *Pseudomonas aeruginosa* phage vB_PaeP_ASP23 and functional analysis of its lysin LysASP and holin HolASP

**DOI:** 10.3389/fmicb.2023.1093668

**Published:** 2023-03-15

**Authors:** Jiaqi Cui, Xiaojie Shi, Xinwei Wang, Huzhi Sun, Yanxin Yan, Feiyang Zhao, Can Zhang, Wenhua Liu, Ling Zou, Lei Han, Qiang Pan, Huiying Ren

**Affiliations:** ^1^College of Veterinary Medicine, Qingdao Agricultural University, Qingdao, Shandong, China; ^2^Qingdao Phagepharm Bio-Tech Co., Ltd., Qingdao, Shandong, China; ^3^College of Chemistry and Pharmaceutical Sciences, Qingdao Agricultural University, Qingdao, Shandong, China

**Keywords:** *Pseudomonas aeruginosa* phage, genome analysis, *lysin*, *holin*, gene expression, phage display technology

## Abstract

In this study, we isolated a lytic *Pseudomonas aeruginosa* phage (vB_PaeP_ASP23) from the sewage of a mink farm, characterized its complete genome and analyzed the function of its putative *lysin* and *holin*. Morphological characterization and genome annotation showed that phage ASP23 belonged to the *Krylovirinae* family genus *Phikmvvirus*, and it had a latent period of 10 min and a burst size of 140 pfu/infected cell. In minks challenged with *P. aeruginosa*, phage ASP23 significantly reduced bacterial counts in the liver, lung, and blood. The whole-genome sequencing showed that its genome was a 42,735-bp linear and double-stranded DNA (dsDNA), with a G + C content of 62.15%. Its genome contained 54 predicted open reading frames (ORFs), 25 of which had known functions. The lysin of phage ASP23 (LysASP), in combination with EDTA, showed high lytic activity against *P. aeruginosa* L64. The holin of phage ASP23 was synthesized by M13 phage display technology, to produce recombinant phages (HolASP). Though HolASP exhibited a narrow lytic spectrum, it was effective against *Staphylococcus aureus* and *Bacillus subtilis*. However, these two bacteria were insensitive to LysASP. The findings highlight the potential of phage ASP23 to be used in the development of new antibacterial agents.

## 1. Introduction

*Pseudomonas aeruginosa* is a Gram-negative opportunistic bacterium that widely exists in soil and water. It is a major pathogen that causes hemorrhagic pneumonia in minks, leading to huge economic losses in the mink farming industry (Qi et al., 2014; Zhao et al., 2018). Minks of all ages are easily affected by bacterial pathogens, especially on hot and humid days from August to November (Zhao et al., 2018). Typical symptoms include cough, nosebleed, and pulmonary hemorrhage (Bai et al., 2019). Antibiotics such as Ceftazidime and Ciprofloxacin are commonly used to treat *P. aeruginosa* infections. However, with the emergence of antibiotic-resistant *P. aeruginosa*, antibiotics has become less effective (Pedersen et al., 2009; Qi et al., 2014). The resistance mechanisms in *P. aeruginosa* are complicated because its genome carries resistance genes which are capable of producing enzymes that can inactivate β-lactams (Moya et al., 2009). Moreover, the active efflux pump system and biofilms are also considered as primary causes for resistance mechanisms (Ziha-Zarifi et al., 1999). All of these factors and mechanisms increase the difficulty of treatments. As of 2018, about 100 million minks have been farmed in Shandong province, which accounts for a large proportion of farmed minks in China. Qian et al. (2020) reported that 20 strains of *P. aeruginosa* isolated from farmed minks in Shandong, China showed multidrug resistance and cross resistance. Furthermore, many clinical cases of antimicrobial resistance have also been reported in the United States, Denmark, and Canada (Nikolaisen et al., 2017; Cormier et al., 2020). The antimicrobial-resistant bacteria detected from the fecal samples belonged to five antimicrobial classes: macrolide–lincosamide–streptogramin B (MLSB; 100% prevalence), TETs (88.1%), β-lactams (71.4%), aminoglycosides (66.7%), and fluoroquinolones (47.6%) (Agga et al., 2021). So it is necessary to develop new antibacterial agents to control *P. aeruginosa* infections in minks. Recent studies have reported the effectiveness of phage therapy for the treatment of *P. aeruginosa* infections in animals (Forti et al., 2018; Lerdsittikul et al., 2022). However, to the best of our knowledge, only Gu et al. (2016) have evaluated the potential of phage therapy in the treatment of hemorrhagic pneumonia in mink by intranasal administration.

Bacteriophages are viruses that can infect most types of bacteria, and lytic phages have been applied to treat bacterial infections (Cisek et al., 2017). With the increasing threat of multi-resistant bacteria, phage therapy is drawing renewed interest in treating bacterial infections (Ryan et al., 2011). In general, a holin-lysin lysis system exists in double-stranded DNA phages to accomplish host lysis (Labrie et al., 2004). During the reproduction cycle, phage-encoded holin accumulates and forms pores in the membrane, leading to an increased access for lysins to degrade peptidoglycan in bacterial cell walls (Kamilla and Jain, 2016). Due to their high hydrolytic activity and pathogen specificity, lysins are considered as prospective antimicrobial agents for the treatment of multi-resistant bacterial infections (Schmelcher et al., 2012). Many studies have demonstrated the *in vitro* therapeutic efficacy of lysins against Gram-positive pathogens (Roach and Donovan, 2015). However, due to the protective outer membranes (OM), the efficacy of lysins against Gram-negative bacteria is limited (Lood et al., 2015). To increase the permeability of the OM, EDTA has been used as an outer membrane permeabilizer (OMP) to disrupt the structure of lipopolysaccharides by removing divalent cations (Briers et al., 2011). In addition, holins are phage-encoded proteins that can effectively kill Gram-positive bacteria extracellularly, including *Streptococcus suis* and *Staphylococcus aureus* (Song et al., 2016).

Compared to phages, holin proteins are safer and more effective in controlling bacterial infections. To obtain high expression of holin proteins, M13 phage display technology is often used. M13 phage is a filamentous virus (about 6.5 nm in diameter and about 900 nm in length), and it has a circular single-stranded DNA wrapped in an outer protein coat composed of about 2,700 copies of helically arranged major coat proteins (pVIII) and five copies of minor coat proteins (pIII, pVI, pVII, and pIX) at both ends of the phage (Ledsgaard et al., 2018). Phage display is a molecular technique based on genetic modification of phage DNA to enable the expression of a peptide/protein on the phage surface in combination with one of the phage coat proteins (Aghebati-Maleki et al., 2016; Sioud, 2019; Jaroszewicz et al., 2022).

In this study, we characterized a lytic phage vB_PaeP_ASP23 (ASP23) isolated from the sewage of a mink farm. Its genome was also sequenced. To understand the lysin-holin lysis system, we further analyzed the *lysin* and *holin* genes of phage ASP23. The findings highlighted the potential application of phage ASP23 in the development of efficient therapeutic agents in the mink farming industry.

## 2. Materials and methods

### 2.1. Ethics statement

Animal experiments performed in this study strictly followed the national guidelines for experimental animal welfare announced by Ministry of Science and Technology of People’s Republic of China in 2006 (Guiding Opinions on Kindly Treating Laboratory Animals^1^) and were approved by the Animal Welfare and Research Ethics Committee at Qingdao Agricultural University, Shandong, China.

### 2.2. Bacterial strains, plasmids, and growth conditions

The bacterial strains and plasmids used in this study are listed in Table 1. All *P. aeruginosa* strains were isolated from minks that died of hemorrhagic pneumonia in Shandong, China. Bacterial strains used for determining antibacterial spectrum were identified by 16S rRNA gene sequencing. In addition, antibiotic susceptibility of *P. aeruginosa* isolates was measured by the Kirby–Bauer disk diffusion method (Forozsh et al., 2012). Bacteria were cultured in Luria Bertani (LB) broth or LB agar (Hopebiol Biotech) at 37°C. The vector pCold TF (N-Trigger factor; Amp^r^; His-Tag, Takara) was used for cloning and expression of the *lysin* gene in *Escherichia coli* BL21 (DE3). The plasmid pCANTAB 5E was used for display and expression of the *holin* gene in M13 phages.

**Table 1:** Bacterial strains, plasmids, and primers used in this study.

Bacterial strains, plasmids, primers	Characteristics, function, or sequence	Source
**Strains**		
*P. aeruginosa* L64	Host for phage ASP23	Our laboratory collection
*P. aeruginosa* PA1-PA11 (11 strains)	Antibacterial spectrum determination	Our laboratory collection
*P. aeruginosa* G3-G10 (8 strains)	Antibacterial spectrum determination	Our laboratory collection
*P. aeruginosa* DL1-DL3 (3 strains)	Antibacterial spectrum determination	Our laboratory collection
*Escherichia coli* E1	Antibacterial spectrum determination	Our laboratory collection
*Salmonella abortus equi* S1	Antibacterial spectrum determination	Our laboratory collection
*Staphylococcus aureus* SA2	Antibacterial spectrum determination	Our laboratory collection
*Bacillus subtilis* A01	Antibacterial spectrum determination	Our laboratory collection
*Proteus mirabilis* P1	Antibacterial spectrum determination	Our laboratory collection
**Plasmids**		
pCold TF	Expression vector; Amp^r^; His-Tag	Takara Biotech
pCold TF-LysASP	Recombinant vector	This study
pCANTAB 5E	Phagemids; Amp^r^; E-Tag	Detai Biotech
**Primers**		
LysASP-F	5′-TAGCATATGGTGAACAAGCCCCT-3′	This study
LysASP-R	5′-TTGGAATTCCTACCACAGCAAGGAC-3′	This study
HolASP-F	5′-ATGATGATTGATACCGCCACCG-3′	This study
HolASP-R	5′-TCACTTCTTGAATCTCCGGCG-3′	This study

### 2.3. Isolation, purification, and characterization of phage ASP23

Phages were isolated from sewage of a mink farm using a *P. aeruginosa* strain (L64) as the host by the double-layer agar method (Lu et al., 2017). The purification and concentration of phage ASP23 were conducted as described previously (Shi et al., 2020). The purified phages were deposited on copper grids and negatively stained with phosphotungstic acid (2% w/v) for 10 min. After drying, the morphology of single phage particles was observed using an HT7700 transmission electron microscope (TEM, Hitachi, Japan) at 80 kV (Zhang et al., 2013).

The one-step growth experiment of phage ASP23 was performed as described previously reported (Shi et al., 2020). The mixtures of phage ASP23 and *P. aeruginosa* strain L64 were collected at different time points, and phage titers were determined immediately using the double-layer agar method. In addition, the host range, thermal stability and pH sensitivity of phage ASP23 were determined as described previously with some modifications (Shi et al., 2020).

### 2.4. *In vivo* therapeutic effect of phage ASP23 in minks infected with *Pseudomonas aeruginosa*

Minks (American mink, *n* = 30, 40–50 days old, average weight = 600 g) were divided into two groups (15 minks per group), including the control group and treatment group. To determine the therapeutic effect of phage ASP23, minks were challenged with *P. aeruginosa* strain L64 (4 × 10^8^ CFU/mink) by intraperitoneal injection. Two hours after challenge, minks in the treatment group received phages by oral gavage (10^10^ pfu/mink). At the same time, minks in the control group received an equal volume of phosphate buffered saline (PBS). Each group contained 15 minks. At different time points (2 h, 5 h, 9 h, 13 h, and 17 h), 3 minks in each group were humanely euthanized with carbon dioxide (Gu et al., 2016). Tissue samples (liver and lung) were collected, weighed, and homogenized in 2 mL sterilized PBS. Blood samples were collected by venipuncture and stored in tubes containing EDTA. Bacterial counts in blood and tissue homogenates were measured by plating serial dilutions on LB agar plates.

### 2.5. Sequencing and bioinformatics analysis of the ASP23 genome

Bacteriophage DNA was prepared using a DNA Viral Genome Extraction Kit (Solarbio) after concentration. The genomic DNA was used to construct a 600-bp insert library using a NEBNext® Ultra™ II DNA Library Prep Kit for Illumina according to the manufacturer’s instruction. The genomic DNA of ASP23 was subjected to high-throughput sequencing using an Illumina HiSeq 2,500 sequencer (San Diego, United States). The complete genome sequence was assembled using CLC Bio (Aarhus, Denmark). Putative open reading frames (ORFs) were predicted using GeneMarkS^2^ and RAST^3^ (Besemer et al., 2001; Aziz et al., 2008). The comparative genomic analysis was performed using BRIG (Alikhan et al., 2011). All predicted ORFs were annotated using BLASTp, and the protein domain analysis was conducted using Pfam.^4^ Putative tRNA genes were identified using tRNAscan-SE. The structural and functional features of putative proteins were analyzed using TransMembrane prediction using Hidden Markov Models^5^ and PredictProtein.^6^ The highly conserved amino acid sequences were used to construct neighbor-joining phylogenetic trees using MEGA 7.

### 2.6. Amino acid sequence analysis, synthesis, and purification of LysASP

The deduced amino acid sequence of LysASP was analyzed using BLASTp, and the sequence alignment was generated using ESPript 3.0.^7^ The sequences similar to LysASP were searched in the Protein Data Bank (PDB), and the structure of LysASP was predicted using Phyre2.^8^

The *lysin* gene was amplified from ASP23 phage genomic DNA using polymerase chain reaction (PCR) with a pair of specific primers containing *Nde*I and *Eco*RI restriction sites. The primers used are listed in Table 1. The PCR product was purified, digested with *Nde*I and *Eco*RI (Takara), and then cloned into the pCold TF expression vector with an N-terminal His6 tag to generate pCold TF-LysASP. The ligation product was transformed into competent cells (*E. coli* strain DH5α, Takara) using the heat shock method (Froger and Hall, 2007). Restriction enzyme digestions and sequencing were used to confirm the cloned fragment. Then the recombinant plasmid was transformed into *E. coli* BL21 (DE3).

The transformed *E. coli* BL21 cells were grown in LB medium containing ampicillin (50 μg/mL, Solarbio) until the OD_600_ reached 0.6 ~ 0.8. The mixed culture was induced with 0.4 mmol/L isopropyl-β-D-thiogalactopyranoside (IPTG) at 16°C for 12 h. Cells were harvested by centrifugation (10,000 × *g*, 15 min) and resuspended in phosphate-buffered solution (PBS, Solarbio), followed by sonication on ice (22 kHz, 45 W, 3 s work, 2 s pause). After centrifugation and filtration, His-tagged proteins were purified using a His-tag Protein Purification kit (Beyotime Biotech, Shanghai, China). The purified proteins were dialyzed against PBS and separated by 12% SDS-PAGE with a molecular weight marker (15–130 kDa, Solarbio). The protein concentration was determined using a BCA Protein Assay Kit (Solarbio, Beijing, China).

### 2.7. Lytic activity of recombinant LysASP against *Pseudomonas aeruginosa* L64

*Pseudomonas aeruginosa* L64 was grown in LB broth to the exponential phase, and 1 mL of culture was pretreated with 2 mM EDTA for 5 min. After centrifugation (10,000 × *g*, 10 min), bacterial cells were washed twice and resuspended in PBS (pH = 7.4). Aliquots (100 μL) of bacterial suspension were added into sterile 96-well plates containing 100 μL of purified LysASP (0.6 mg/mL) per well, followed by incubation at 37°C. The OD_600_ values were measured by a microplate reader at different time points. Simultaneously, the EDTA-pretreated bacterial suspension was added in PBS without LysASP and used as negative control. In the end, the colony-forming units (CFU) were determined. The experiment was performed in triplicate.

### 2.8. Determination of the lytic range of LysASP

The lytic activity of LysASP against *P. aeruginosa* (PA1-PA11), *E. coli* (E1), *Salmonella abortus equi* (S1) and *S. aureus* (SA2) was determined based on OD_600_ of bacterial culture measured by a microplate reader as mentioned above. *P. aeruginosa* strain L64 and PBS were used as positive and negative controls, respectively. All bacteria strains are listed in Table 1. The experiment was performed in triplicate.

### 2.9. Expression of *Holin* by M13 phage display technology

The deduced amino acid sequence of *holin* was analyzed by BLASTp, and putative hydrophobic transmembrane domains were predicted using TMHMM 2.0.^9^ The *holin* gene was amplified from phage ASP23 genomic DNA using PCR with a pair of specific primers containing *Sfi*I and *Not*I restriction sites. The primers used are listed in Table 1. The PCR product was purified, digested with *Sfi*I/*Not*I (Takara), and cloned into the pCANTAB 5E phagemids to generate pCANTAB 5E-holin, in which a C-terminal E-tag was used. The ligation product was transformed into competent cells (*E. coli* strain TG1) using the heat shock method. Restriction enzyme digestions and sequencing were used to confirm the cloned fragment.

To produce recombinant fusion M13 phages, a single colony of pCANTAB 5E-holin transformed *E. coli* TG1 cells was grown in 2 × YT medium containing 2% glucose and 50 μg/mL ampicillin at 37°C with shaking (220 rpm) until the OD_600_ reached 0.5–0.7. Then, helper phage M13KO7 was added in the medium at a multiplicity of infection of about 1:50, followed by incubation for 35 min. After centrifugation, cells were re-suspended in fresh 2 × YT medium containing 25 μg/mL kanamycin and cultivated for at 37°C with shaking (220 rpm) for 20 h. After centrifugation (13,000 × *g* for 20 min, 4°C), phages were concentrated and precipitated by adding 1/5 (v/v) of PEG/NaCl solution in the supernatant. After incubation on ice for 12 h, the mixture was centrifuged, and the pellet was resuspended in 800 μL PBS (0.01 mM; pH 7.4), followed by incubation at 4°C for 12 h. Finally, after centrifugation, the supernatant containing M13 phages was collected and stored at 4°C (Tanaka et al., 1995). The holin protein of recombinant phages was identified using mass spectrometry (MS)-based shotgun proteomics.

### 2.10. Determination of the lytic range of HolASP

To determine the lytic range of HolASP (recombinant phages), which was made with M13KO7 displaying *holin* of ASP23, six strains of bacteria were tested (Table 1), including two Gram-positive bacteria (*Staphylococcus aureus* SA2 and *Bacillus subtilis* A01) and four Gram-negative bacteria (*P. aeruginosa* L64, *E. coli* E1, *S. abortus equi* S1, and *Proteus mirabilis* P1). Briefly, qualitative filter papers were placed onto freshly seeded lawns of bacteria, on which 10 μL HolASP (at a final concentration of 10^8^ virions/mL) was spotted (Song et al., 2021). After incubation at 37°C for 12 h, the production of transparent antibacterial rings was assessed. Simultaneously, the mixed solution of PBS and M13KO7 was used as a negative control to exclude the effect of M13KO7 on bacteria.

### 2.11. Measurement of antimicrobial activity of HolASP

The six strains of bacteria mentioned above were used as indicator strains to determine the antimicrobial activity of recombinant phages (HolASP). Bacterial cells were washed and resuspended with sterile phosphate-buffered saline (PBS) to 5 × 10^7^ CFU/mL (Song et al., 2021). HolASP (at the final concentration of 10^8^ virions/mL) was added to the bacterial suspension, and the mixture was incubated at 37°C for 12 h. The antibacterial activity was expressed as CFU reduction following treatment. As a negative control, the bacterial strains were treated with elution buffer (PBS, 0.01 mM; pH 7.4) under the same conditions.

### 2.12. Genome sequence accession number

The complete genome sequence of phage ASP23 was deposited in GenBank under the accession number MN602045.

### 2.13. Statistical analyses

The experiments, including the one-step growth curve, temperature sensitivity and pH stability of ASP23, lytic activity of LysASP and antibacterial activity of HolASP, were performed in triplicate. The results were presented as means ± standard deviation (SD). Student’s t-test and analysis of variance were used to compare the differences between the numbers of bacteria in different organs using GraphPad Prism 6.0 (GraphPad, CA, United States). Differences were considered statistically significant when *p* < 0.05.

## 3. Results

### 3.1. Isolation and characterization of phage ASP23

**Figure 1:**
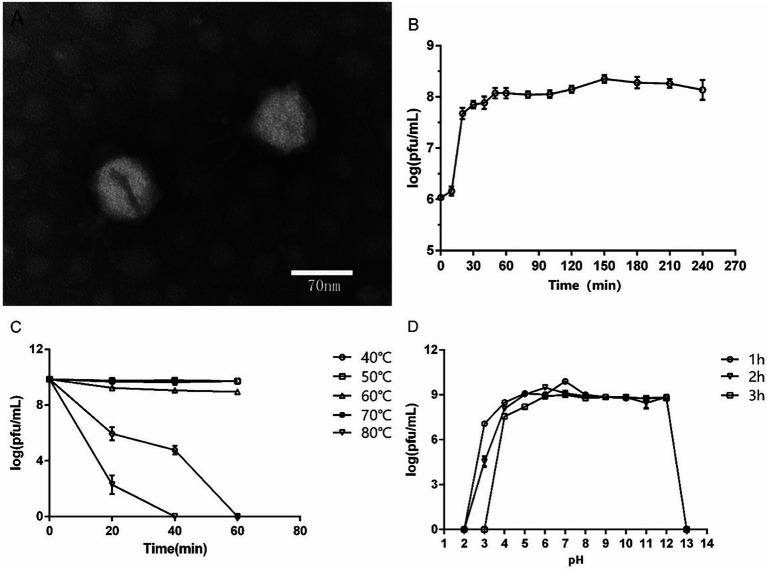
Biological characteristics of phage ASP23. **(A)** TEM image of phage ASP23. Phage ASP23 had an icosahedral head of 70 nm in diameter and a short tail with 40 nm in length. **(B)** One-step growth curve of ASP23. The latent period and burst size were 10 min and 140 pfu/infected cell. **(C)** Temperature sensitivity of ASP23. The phage titers were no significant differences at 40°C ~ 60°C. **(D)** pH stability of ASP23. Phage ASP23 was stable over a broad range of pH (5–12). Data are expressed as mean ± SD (*n* = 3).

A *P. aeruginosa* phage was isolated from sewage and named as vB_PaeP_ASP23 (ASP23). The TEM image showed that phage ASP23 had an icosahedral head of 70 nm in diameter and a tail of 40 nm in length (Figure 1A), suggesting that the phage ASP23 belonged to the phiKMV-like phages genus. The one-step growth curve of ASP23 revealed that the latent period and lysis period were 10 and 45 min, respectively, and the average burst size was 140 pfu/infected cell (Figure 1B). The thermal stability test showed that ASP23 was stable below 60°C, but it was completely inactivated at 80°C after 40 min (Figure 1C). In addition, the titer of ASP23 could be maintained above 10^6^ PFU/mL within 20 min at 70°C. ASP23 could maintain high activity (10^9^ PFU/mL) over a broad range of pHs (5–12) for at least 3 h (Figure 1D), but it was inactivated at extreme pHs (below pH 4 or above pH 12). ASP23 exhibited lytic activity and lyzed 68% (15/22) of the *P. aeruginosa* strains (Table 2).

**Table 2:** Antibacterial activities of phage ASP23 and LysASP toward different species of bacteria.

Strain	Antibiotic susceptibility^a^				Lytic activity of ASP23	Lytic activity of LysASP^b^
	PIP	CAZ	OFX	CIP		
**Gram-negative strains**						
*P. aeruginosa* L64 (host)	R	R	S	R	+	+
*P. aeruginosa* PA1	R	I	R	I	+	+
*P. aeruginosa* PA2	R	R	S	I	+	+
*P. aeruginosa* PA3	R	R	I	I	+	+
*P. aeruginosa* PA4	R	R	S	I	−	−
*P. aeruginosa* PA5	R	R	R	R	−	−
*P. aeruginosa* PA6	R	R	I	R	+	+
*P. aeruginosa* PA7	R	I	I	R	+	+
*P. aeruginosa* PA8	R	R	S	S	+	+
*P. aeruginosa* PA9	S	R	I	R	+	+
*P. aeruginosa* PA10	I	R	R	R	+	+
*P. aeruginosa* PA11	S	R	R	R	+	+
*P. aeruginosa* G3	R	R	S	I	+	+
*P. aeruginosa* G4	S	I	R	R	+	+
*P. aeruginosa* G5	R	R	I	I	+	+
*P. aeruginosa* G6	R	R	S	R	−	−
*P. aeruginosa* G8	I	R	R	R	+	+
*P. aeruginosa* G9	S	R	I	R	+	+
*P. aeruginosa* G10	R	I	I	R	−	−
*P. aeruginosa* DL1	S	R	R	R	−	−
*P. aeruginosa* DL2	R	R	S	I	−	−
*P. aeruginosa* DL3	I	R	R	S	−	−
*E. coli* E1	−	−	−	−	−	−
*S. abortus equi* S1	−	−	−	−	−	−
Gram-positive strain						
*S. aureus* SA2	−	−	−	−	−	−

^a^S, susceptible; I, intermediate; R, resistant; −, not test; ^b^Gram-negative bacteria were pretreated with EDTA. +, lysis; −, no lysis. PIP, Piperacillin; CAZ, Ceftazidime; OFX, Ofloxacin; and CIP, Ciprofloxacin.

### 3.2. Therapeutic effect of phage ASP23 in minks challenged with *Pseudomonas aeruginosa* L64

**Figure 2:**
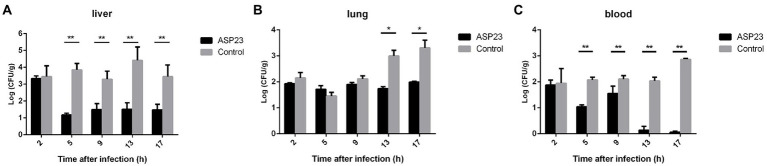
Bacterial counts in two tissues and blood of minks treated with phage ASP23 after they were challenged with *P. aeruginosa* L64 for 2 h. **(A)** liver, **(B)** lung, and **(C)** blood. Data are expressed as mean ± SD (*n* = 3). *p < 0.05, **p < 0.01.

To determine the therapeutic effect of phage ASP23 against infection caused by *P. aeruginosa* L64, we treated minks with phage ASP23 after they were challenged with *P. aeruginosa* L64 for 2 h. As shown in Figure 2, phage ASP23 significantly reduced bacterial counts in the liver and blood early at 5 h after treatment, and bacterial count in the lung decreased significantly at 13 h after treatment. The result indicated that phage ASP23 had a therapeutic effect in minks against *P. aeruginosa* L64.

### 3.3. Complete genome analysis of phage ASP23

**Figure 3:**
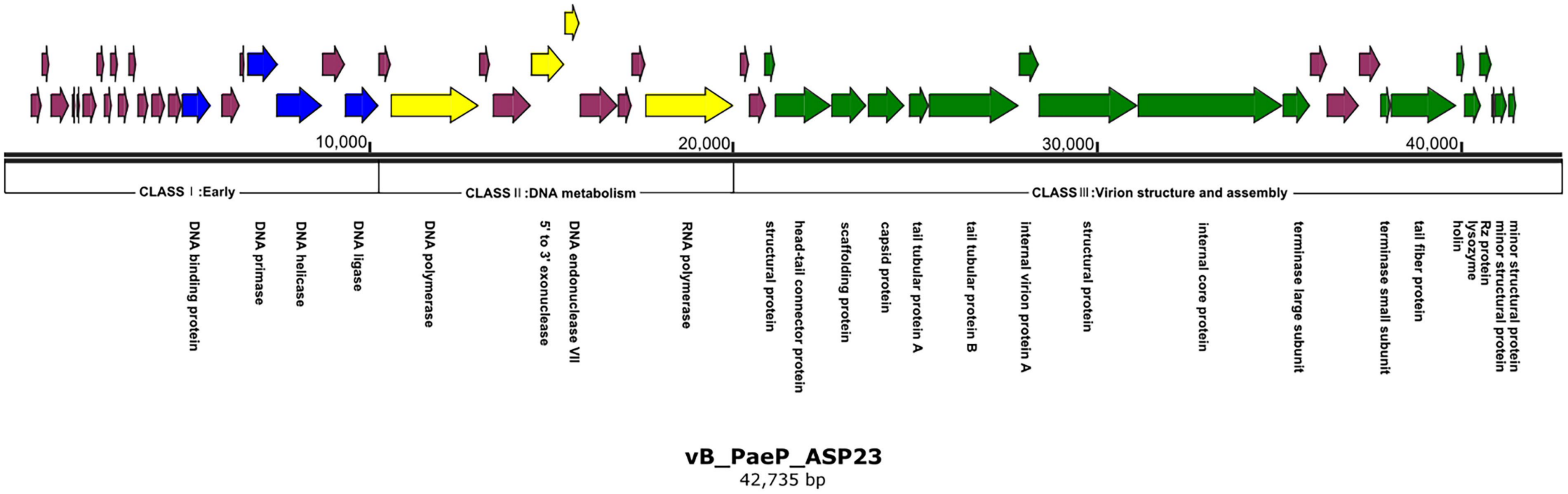
Genome map of phage ASP23. Nucleotide sequences were analyzed using SnapGene and predicted open reading frames are denoted by arrows. Genes were classified into different clusters: early genes (blue), genes involved in DNA metabolism (yellow), genes involved in virion structure and assembly (green), genes encoding hypothetical proteins (purple).

**Figure 4:**
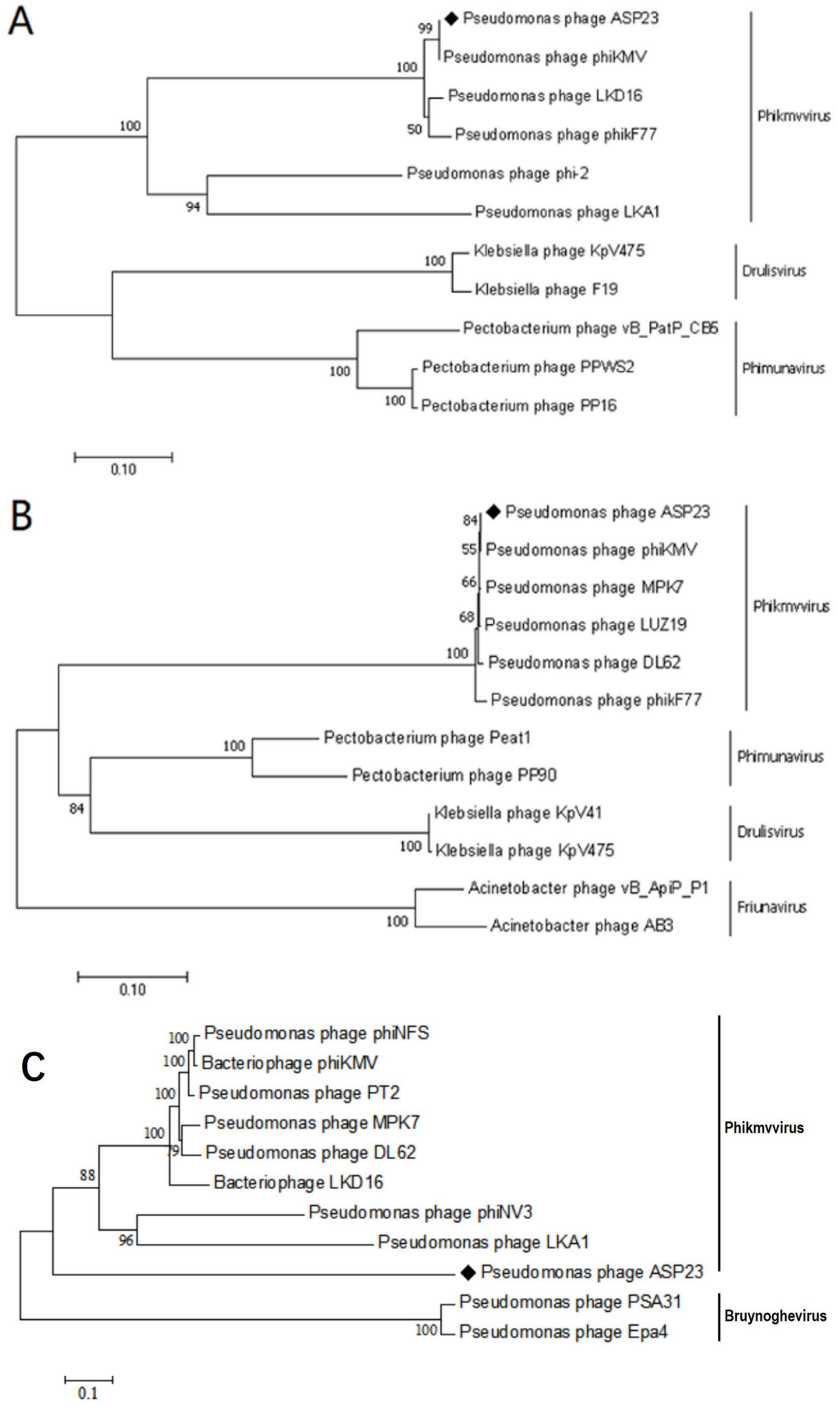
Neighbor-joining phylogenetic trees for the amino acid sequences of **(A)** Major capsid protein **(B)** DNA polymerase and **(C)** Whole genome sequence showing the evolutionary relationships between phage ASP23 and other known phages.

The ASP23 genome was 42,735 bp in size and had an overall G + C content of 62.15%, which was similar to lytic phages of the genus *phikmvvirus*, such as Lx18 (100%, GenBank Accession No. MN692672.2), φKMV (98.62%, GenBank Accession No. AJ505558.1) and phiNFS (GenBank Accession No. NC_047852.1). By calculating the average nucleotide identity (ANI) value and the *in silico* DNA–DNA hybridization (*is*DDH) identity, we found that the ANI and *is*DDH values between phage ASP23 and Lx18 were 100%, the ANI and *is*DDH values between ASP23 and phiKMV were 98.08 and 76%, respectively, and the ANI and *is*DDH values between ASP23 and phiNFS were 98.22 and 81.3%, respectively. A total of 54 putative open reading frames (ORFs) longer than 100 bp were predicted, and no ORFs associated with drug resistance or lysogenization were identified (Supplementary Table S1). Among them, 45 ORFs (83.33%) had an ATG start codon, five ORFs had a GTG start codon, and four ORFs carried a TTG start codon. All predicted ORFs were located on the sense strand of the genome. BLASTp analysis revealed that 25 ORFs were annotated to encode proteins of known function. Similar to phage φKMV, the genes of ASP23 could be classified into three clusters: early genes (class I), genes involved in DNA metabolism (class II), and genes encoding structural and lysis proteins (class III) (Figure 3). To investigate the evolutionary relationship between ASP23 and other phages, whole genome sequence, major capsid protein and DNA polymerase were chosen for the construction of phylogenetic trees. As shown in Figure 4, phage ASP23 was clustered with phage φKMV and located near the members of the genus *Phikmvvirus*, confirming their close evolutionary relationship. Thus, phage ASP23 could be classified as a member of the genus *Phikmvvirus* in the family *Krylovirinae*.

Similar to *Phikmvvirus* phages, the early region (ORF1-ORF21) ended after the DNA ligase gene, and these genes included DNA-binding protein (ORF15), DNA primase (ORF18), DNA helicase (ORF19), and DNA ligase (ORF21). The amino acid sequence of DNA ligase showed high identity with φKMV_gp17 and contained a conserved domain (PHA00454). Four ORFs involved in DNA metabolism were identified in the genome of phage ASP23. ORF23 appeared to encode DNA polymerase, and its C-terminal region contained a DNA_pol_A domain (pfam00476). ORF26 and ORF27 were predicted to encode exonuclease and DNA endonuclease VII, respectively. ORF31 was predicted to encode RNA polymerase, and it was not located in the early gene region but at the end of the DNA metabolism region. The structural module of ASP23 was comprised of twelve ORFs, which encoded structural and packaging proteins, capsid proteins, and tail-related proteins. Generally, the terminase is composed of two subunits (big subunit and small subunit) which are involved in DNA packaging (Catalano, 2000). In the ASP23 genome, ORF47 and ORF48 encoded small subunit and big subunit, respectively. For the lysis system of ASP23, ORF49 and ORF50 encoded *holin* and *lysin*, respectively. ORF49 shared sequence homology with a putative *holin* gene in phage phikF77, moreover, its small size and genome location showed that it might encode a *holin*. Furthermore, ORF49 was a membrane protein with a putative hydrophobic transmembrane domain (TMD) and had a hydrophilic C-terminus with several charged amino acids, which should be classified into class II holins.

### 3.4. Identification and characterization of LysASP

The 483-bp *lysin* gene of phage ASP23, named as LysASP, contained 160 amino acid residues. Three sequences with high identity to LysASP were selected, and multiple sequence alignments were performed. The Pfam database revealed that it contained a lysozyme-like domain between residues 34 and 141 of LysASP (Supplementary Figure S1A). An alignment of LysASP with three phage lysins showed high similarity in the conserved domain, however, three non-conserved residues (amino acids 61, 64, and 76) were found in the conserved domain (Supplementary Figure S1B). Based on the sequence searches in PDB, LysASP shared the highest identity (30.6%, 59/196) with the Muramidase domain of SpmX (Supplementary Figure S2A; Randich et al., 2019). Furthermore, the structure of LysASP was predicted using Phyre2, and the confidence of the model was 100.0% (Supplementary Figure S2B). Although the predicted backbone structure of the LysASP shared high similarity with SpmX muramidase domain, their surface representations were different (Supplementary Figures S2C,D).

### 3.5. Synthesis and purification of LysASP

The LysASP gene was successfully amplified from ASP23 genomic DNA and was cloned into the *Nde*I/*Eco*RI sites of the pCold TF expression vector. The recombinant plasmid was confirmed by DNA sequencing. After induction with IPTG, the expression of the fusion protein was detected by SDS-PAGE (Supplementary Figure S3). As expected, the size of LysASP was ~69 kDa and most of the recombinant protein was found to be in the soluble fraction after sonication.

### 3.6. Lytic activity of LysASP

**Figure 5:**
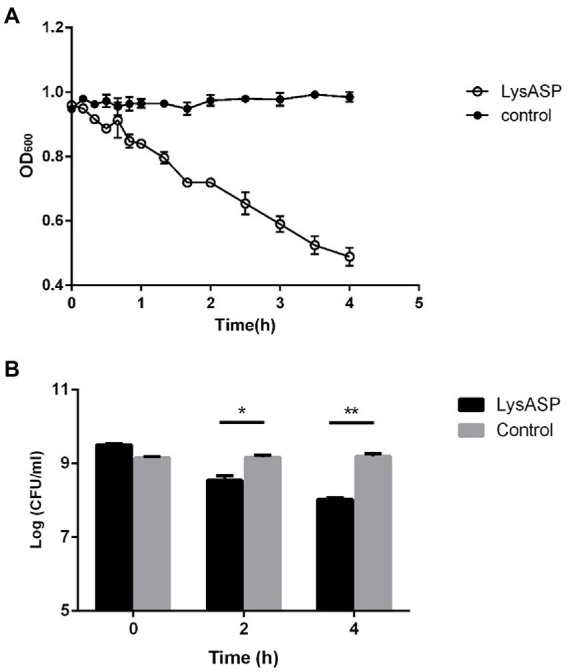
Lytic activity of purified LysASP against *P. aeruginosa* L64. **(A)** Changes in OD_600_ of bacterial culture following LysASP treatment, **(B)** Bacterial counts at different time points following LysASP treatment. Data are expressed as mean ± SD (*n* = 3). *p < 0.05, **p < 0.01.

The lytic activity of LysASP was determined against bacterial cells. As shown in Figure 5, the OD_600_ values of bacterial cells treated with LysASP decreased remarkably from 0.95 to 0.48, whereas the OD_600_ values of bacterial cells in the control group remained constant. Besides, LysASP treatment significantly decreased bacterial counts of *P. aeruginosa* L64 at 2 and 4 h, compared to the control group. These results showed that, in combination with EDTA, LysASP had lytic activity against *P. aeruginosa L16.*

### 3.7. Antibacterial spectrum of LysASP

To test the lytic range of LysASP, we treated Gram-positive bacteria (*S. aureus* strain SA2) and Gram-negative bacteria (*P. aeruginosa* PA1-PA11, *P. aeruginosa* G3-G10, *P. aeruginosa* DL1-DL3, *E. coli* E1, and *S. abortus equi* S1) with LysASP. The results showed that LysASP exhibited antibacterial activity toward 15 strains of *P. aeruginosa*, but it could not lyse other three bacterial species, which was the same as the antibacterial spectrum of phage ASP23 (Table 2).

### 3.8. Prediction and expression of *Holin*

TMHMM analysis showed that *holin* of phage ASP23 is a membrane protein with typical *holin* traits (Supplementary Figure S4A), which consisted of 66 amino acids (aa). And disulfide bond, signal peptide and coiled helix were not predicted in *holin*. *Holin* of phage ASP23 had a hydrophobic TMD at the N-terminus and multiple positively charged amino acids at the hydrophilic C-terminus. Since *holin* of phage ASP23 shared structural characteristics with class II holins, it could be considered as a member of the class II family (Reddy and Saier, 2013).

The *holin* gene was successfully amplified from ASP23 genomic DNA and was cloned into the *Sfi*I/*Not*I sites of the pCANTAB 5E phagemids. The recombinant phagemids were confirmed by DNA sequencing. The results of SHOTGUN proteomics indicated that holin protein was successfully displayed on the surface of recombinant phage. The titer of rescued recombinant phage was 6 × 10^8^ virions/mL (Supplementary Figure S4B).

### 3.9. Antibacterial activity of HolASP

**Figure 6:**
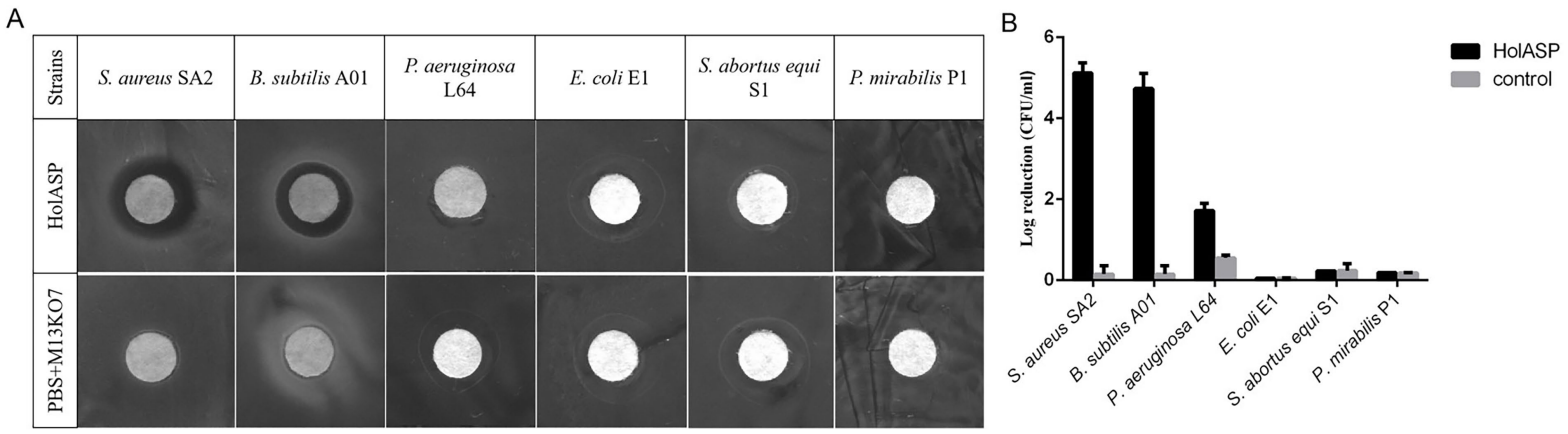
The antibacterial activity of recombinant phage HolASP. **(A)** Images of plaques formed on the lawns of different species of bacteria produced by HolASP. Plate lytic assay. The antibacterial activity of HolASP (10^8^ virions/mL) against different species of bacteria was tested using plate assays. The mixed solution of PBS and M13KO7 was used as a negative control. **(B)** Antibacterial activity of HolASP against different species of bacteria. The bacterial suspension (5 × 10^7^ CFU/mL) were exposed to HolASP (10^8^ virions/mL) at 37°C for 12 h. Antibacterial activity *in vitro* was expressed as CFU reduction. The bacterial suspension without treatment with HolASP was used as control. Data are represented as mean ± SD (*n* = 3). CFU, colony-forming unit; SD, standard deviation.

The antibacterial abilities of HolASP are shown in Figure 6. HolASP produced clear rings on bacterial lawns of *Staphylococcus aureus* SA2 and *Bacillus subtilis* A01. However, HolASP had no lytic ability against three of the four Gram-negative bacteria, including *E. coli* E1, *S. abortus equi* S1 and *P. mirabilis* P1. Log-phase cultures of six tested strains were exposed to HolASP (final concentration, 6 × 10^8^ virions/mL) for 12 h. HolASP treatment resulted in log_10_ CFU reductions of two Gram-positive bacteria (5.3-log10 CFU reduction of *S. aureus* SA2 and 4.48-log10 CFU reduction of *B. subtilis* A01), relative to the controls. Furthermore, HolASP showed slight lytic ability against its host bacterium *P. aeruginosa* L64 (1.6-log10 CFU reduction of L64). The results indicated that HolASP only had efficient lytic ability against Gram-positive bacteria.

## 4. Discussion

*Pseudomonas aeruginosa* is a common opportunistic pathogen that can rapidly develop resistance to different types of antibiotics, which makes it difficult to treat *P. aeruginosa* infections (Lu et al., 2013; Qian et al., 2020). In the present study, a new phage ASP23 was isolated from sewage of a mink farm using a mink-derived *P. aeruginosa* strain as host. The biological properties of phage ASP23 were characterized. Due to its short latent period and lack of lysogenesis-related genes, phage ASP23 met the prerequisites for phage therapy. Moreover, phage ASP23 exhibited high resistance to high temperatures and extreme acidic/alkaline environment, which is beneficial to its storage and utilization. More importantly, the therapeutic study showed the ability of phage ASP23 to control *P. aeruginosa* infections in minks. Due to the rapid spread of *P. aeruginosa* after intraperitoneal injection, we did not find significant differences in bacterial counts between phage-treated group and control group at 2 h following treatment. It was reported that a single intranasal dosage of phage YH30 (1 × 10^8^ PFU/mL) can protect 100% minks against hemorrhagic pneumonia 2 h after *P. aeruginosa* challenge (Gu et al., 2016). By contrast, our study demonstrated that phage ASP23 also had an effective therapeutic effect through oral gavage rather than intranasal administration.

The antibiotic resistance in *P. aeruginosa* is rapidly increasing, and it is important to develop therapeutic agents for bacterial infections. In clinical application, quinolones and β-Lactam antibiotics are often used to treat *P. aeruginosa* infections. In our study, all 22 strains of *P. aeruginosa* showed drug resistance to commonly used antibiotics. More importantly, 18 of the 22 isolates (81.81%) showed resistance to Ceftazidime, and 59.09% of the isolates were resistant to Ciprofloxacin. Phage-encoded lysins have high potential to be used as new alternatives to antibiotics. To date, there are many lysins known to target *P. aeruginosa.* In general, due to the presence of the outer membrane in Gram-negative bacteria, exogenous lysins could not access the peptidoglycan layer (Fischetti, 2010). On the other hand, outer membranes can be destabilized by many methods, such as outer membrane permeabilizer (EDTA, citric acid, etc.) and high hydrostatic pressure (50 to 200 MPa) (Briers et al., 2008, 2011).

Many studies have shown that lysins can control Gram-negative bacterial infections when the outer membrane is destabilized. Briers et al. (2011) reported that the combination of *lysin* EL188 and EDTA showed antimicrobial activity against *P. aeruginosa* and reduced bacterial count up to 4 log units in 30 min. In this study, we used EDTA to destabilize the outer membrane of host cells, and demonstrated that the recombinant LysASP in combination with EDTA could remarkably inhibit the growth of *P. aeruginosa* L64. Furthermore, the combination of LysASP and EDTA exerted lytic activity against other nine *P. aeruginosa* strains. Similarly, Lai et al. (2011) found that the lysozyme (LysAB2) of phage φAB2 had a lytic effect on EDTA-pretreated bacterial cells. However, LysASP did not exhibit lytic activity against other Gram-negative and Gram-positive bacterial strains. This was consistent with a previous study that lysin possesses specificity at the host level (Loessner, 2005). From a safety point of view, it is expected that phage lysins can specifically kill target pathogens without destroying commensal microflora (Nelson et al., 2012). The result indicated that LysASP had relatively higher specificity for *P. aeruginosa* than other tested bacteria. However, LysASP could not lyse bacterial cells without OMPs.

Holins have been identified in a wide variety of phages. Holins possess some common characteristics. For example, holins have at least one transmembrane α-helical region that has a highly charged hydrophilic C-terminal domain and is crucial for their functionality. A gene close to the *endolysin* gene encodes the majority of holins. During the late infection period, phage-encoded holin proteins can accumulate in the cytoplasmic membrane before triggering; when the concentration of holin proteins reaches a threshold, micronscale holes will be formed, which allow the soluble endolysins to be released from the cytoplasm to reach the peptidoglycan in the cell wall. The function of holins is associated with a collapse of the membrane potential and permeabilization of the membrane (Young, 1992). In this respect, holins have the potential to be used as therapeutic agents in their own right (Donovan, 2007). In this study, we found that HolASP had efficient lytic ability only against Gram-positive bacteria, such as *B. subtilis* A01 and *S. aureus* SA2. Similarly, it has been reported that HolSMP, the holin protein of phage SMP, shows efficient bactericidal ability against Gram-positive bacteria only, such as *S. aureus* and *B. subtilis* (Shi et al., 2012). The holin-like protein from *B. licheniformis* has also been reported to be effective at killing several types of Gram-positive bacteria, such as methicillin-resistant *S. aureus* (MRSA) and *Micrococcus luteus* (Anthony et al., 2010). Compared with *Streptococcus* spp., *P. aeruginosa* and *E. coli*, many *S. aureus* strains have no capsule but a slime layer, so it is possible that it might be easier for holin to target the membrane of *S. aureus.* However, the exact antimicrobial mechanism of holins needs to be elucidated in the future.

Phage display is a powerful method for selecting and engineering polypeptides with desired peptide-binding specificity, and it is achieved by fusing polypeptide libraries to phage coat proteins (Smith and Petrenko, 1997; Sidhu, 2000; Hamzeh-Mivehroud et al., 2013; Hess and Jewell, 2020; Jaroszewicz et al., 2022). This method relies on the fact that if the gene fragment encoding the polypeptide is fused with the M13 coat protein gene, these “fusion genes” can be integrated into phage particles, and the surface of phage particles also shows heterologous proteins (Sidhu, 2001). In this way, a physical linkage is established between phenotype and genotype. Peptides fused to M13 coat proteins will be displayed on the surface of phage particles, given that these fusion proteins can successfully pass through the assembly device and be integrated into assembled phages without affecting their viability. However, the utility of a phage-displayed library depends on the display quality, because a particular library member is only selectable when DNA-encoded polypeptides are efficiently displayed on phage surface. In this study, we integrated the *holin* gene with 66 amino acid residues into the pIII protein of M13 phage, and the concentration of recombinant phage after rescue was 6 × 10^8^ virions/mL. Since the Phagemid display system is univalent phage display and the concentration of the tested strain is 5 × 10^7^ CFU/mL, it can be concluded that 12 protein molecules can lyse one bacterial cell. Therefore, this method can be applied to compare protein lysis ability. In addition, T4 phage is a virulent coliphage that can lyse Gram-negative bacteria. In this case, since HolASP can only lyse Gram-positive bacteria, it is possible for holin to be displayed on T4 phage, which might greatly expanded its lytic spectrum.

## 5. Conclusion

In this study, we analyzed the complete genome of a lytic *P. aeruginosa* phage isolated from sewage of a mink farm. This phage belonged to the genus *Phikmvvirus* and was named vB_PaeP_ASP23. We performed functional analysis of its putative *lysin* and *holin*. The *lysin* gene was designated as LysASP, functional analysis revealed that LysASP had efficient bactericidal ability against Gram-negative bacteria. Holin protein synthesized by M13 phage display showed efficient lytic activity only against Gram-positive bacteria. Phage ASP23, LysASP and recombinant phage HolASP could be used as potential candidates for the development of therapeutic/antibacterial agents against bacterial infections.

## Data availability statement

The datasets presented in this study can be found in online repositories. The names of the repository/repositories and accession number(s) can be found in the article/Supplementary material.

## Author contributions

HR designed the study and analyzed the data with QP. JC and XS contributed to the study execution with help from XW, WL, CZ, and LZ on bacteria collection and reagents. HS, YY, and LH conducted animal experiments. JC drafted the manuscript. All authors were responsible for data integrity and accuracy of the analysis and approved the final version of the manuscript.

## Funding

This work was funded by grants from the Donkey Industry Innovation Team Program of Modern Agricultural Technology System from Shandong Province, China (SDAIT-27-04) and the Youth Innovation Team Project for Talent Introduction and Cultivation for the universities in Shandong province, China.

## Conflict of interest

HS, YY, FZ, and QP were employed by Qingdao Phagepharm Bio-tech Co., Ltd.

The remaining authors declare that the research was conducted in the absence of any commercial or financial relationships that could be construed as a potential conflict of interest.

## Publisher’s note

All claims expressed in this article are solely those of the authors and do not necessarily represent those of their affiliated organizations, or those of the publisher, the editors and the reviewers. Any product that may be evaluated in this article, or claim that may be made by its manufacturer, is not guaranteed or endorsed by the publisher.

## Footnotes

^1^ https://www.most.gov.cn/xxgk/xinxifenlei/fdzdgknr/fgzc/gfxwj/gfxwj2010before/201712/t20171222_137025.html

^2^ http://exon.gatech.edu/Genemark/genemarks.cgi

^3^ http://rast.nmpdr.org

^4^ http://pfam.xfam.org/

^5^ http://www.cbs.dtu.dk/services/TMHMM/

^6^ http://www.predictprotein.org

^7^ http://espript.ibcp.fr/ESPript/ESPript/

^8^ http://www.sbg.bio.ic.ac.uk/phyre2/html/page.cgi?id=index

^9^ http://www.cbs.dtu.dk/services/TMHMM/
