# Supplementary material for: Characterization of a lytic Pseudomonas aeruginosa phage vB_PaeP_ASP23 and functional analysis of its lysin LysASP and holin HolASP
Source: Front Microbiol. 2023 Mar 15;14:1093668. doi: 10.3389/fmicb.2023.1093668 (PMC10045481; doi:10.3389/fmicb.2023.1093668)
Supplement: Supplementary file 1 [file Data_Sheet_1.docx]

Supplementary Material

# Supplementary Figures and Tables

## Supplementary Figures


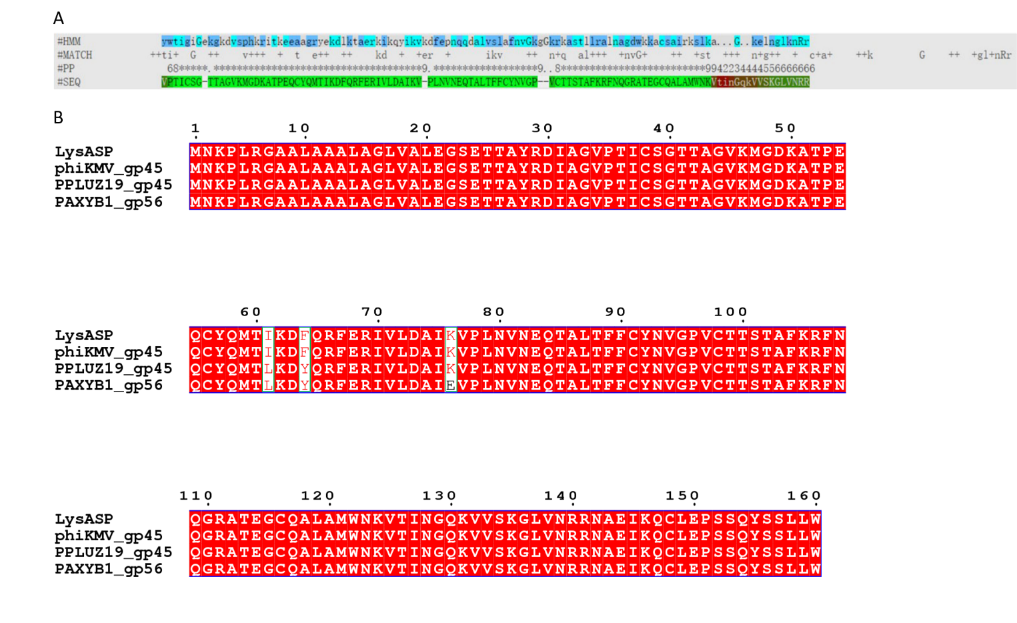


**Supplementary Figure 1.** Sequence analysis of LysASP. **(A)** Visualization of the conserved domain between residues 34 and 141 of LysASP. **(B)** Sequence analysis of LysASP and homologous proteins, and three boxes indicate the non-conserved residues.


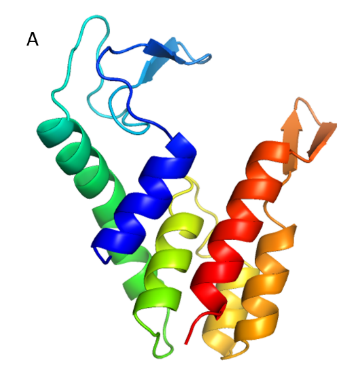

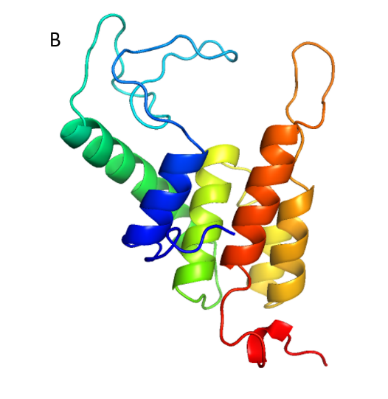

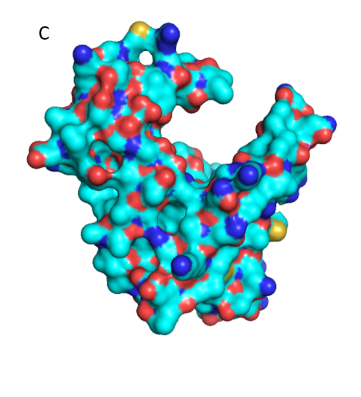

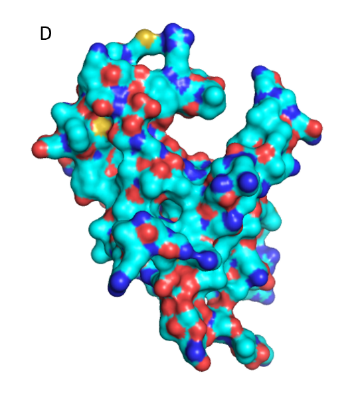


**Supplementary Figure 2.** The structural model of LysASP. **(A)** The structural model of the Muramidase domain of SpmX (PDB ID: 6H9D), **(B)** The structure model of LysASP predicted using Phyre2, **(C)** Surface representation of the groove in Muramidase domain of SpmX, and **(D)** Surface representation of the groove in LysASP.


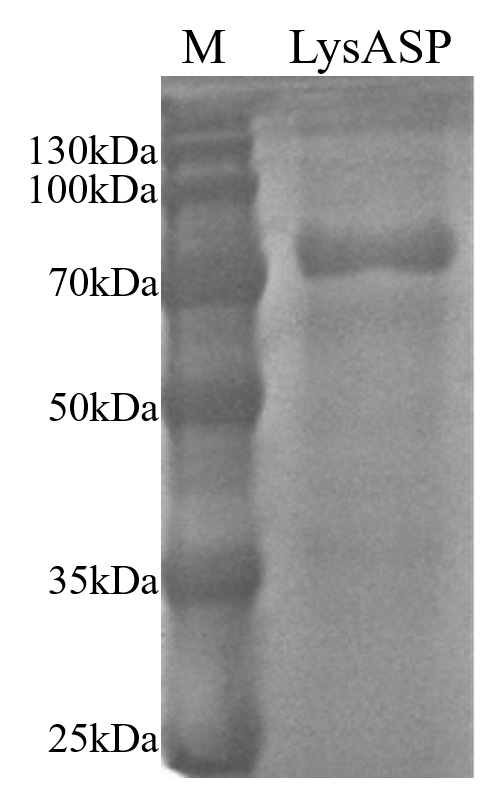


**Supplementary Figure 3.** Protein expression of LysASP. *E. coli* BL21 harboring the recombinant plasmids (pCold TF-LysASP) were induced with 0.4 mmol/L IPTG at 16 °C for 12 h. After ultrasonic purification, the expressed proteins in the supernatant were collected and detected by SDS-PAGE.


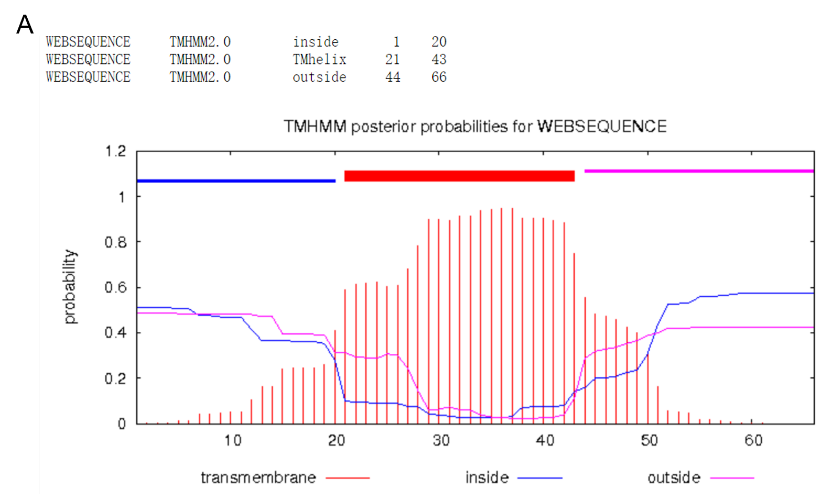

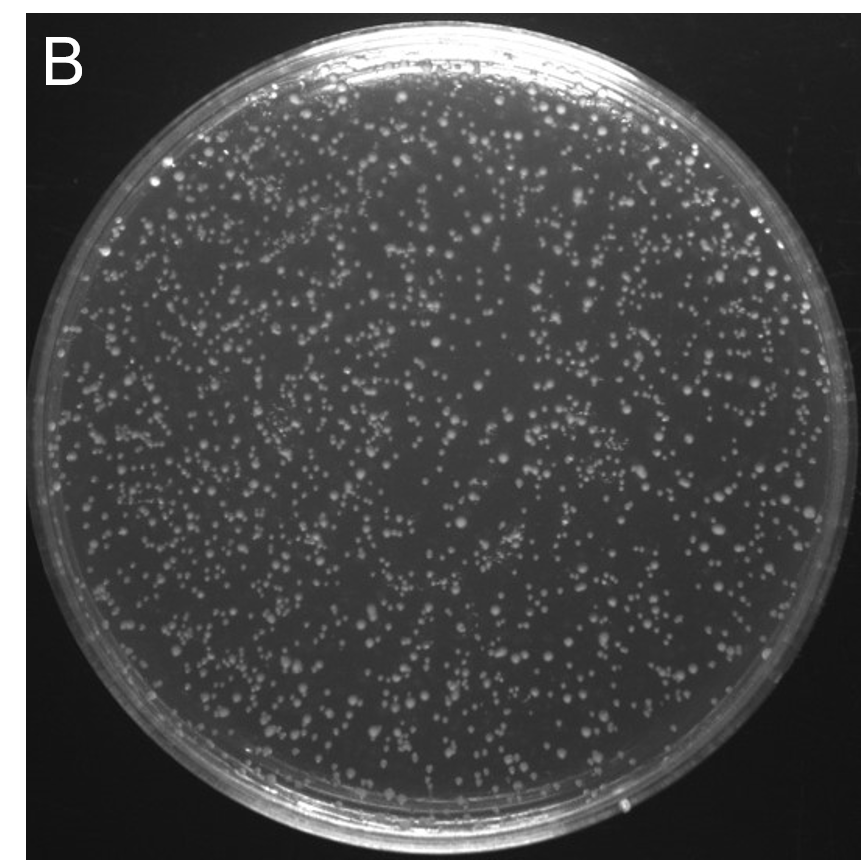


**Supplementary Figure 4.** Prediction and expression of HolASP. **(A)** Prediction of the putative hydrophobic transmembrane domains (TMDs) of HolASP using TMHMM 2.0, and **(B)** the titer of recombinant phages. Recombinant phages were incubated with equal volume *E. coli* TG1 and then the mixture was cultured on LB agar containing ampicillin (50 μg/ml, Solarbio) at 37 °C for 12 h. The titer of recombinant phages was expressed as the number of colonies.

## Supplementary Table

**Table S1** Genome annotation of phage ASP23

| ORFs | Start | Stop | Strand | Size(aa) | Function | Best-match BLASTp Result | Identities | E-values | Accession |
| --- | --- | --- | --- | --- | --- | --- | --- | --- | --- |
| ORF1 | 719 | 1003 | + | 94 | hypothetical protein phiKMVp01 | [Pseudomonas phage phiKMV] | 100% | 2.00E-62 | NP_877440.1 |
| ORF2 | 1003 | 1230 | + | 75 | hypothetical protein phiKMVp02 | [Pseudomonas phage phiKMV] | 100% | 3.00E-46 | NP_877441.1 |
| ORF3 | 1241 | 1780 | + | 179 | hypothetical protein PT2_gp04 | [Pseudomonas phage PT2] | 100% | 2.00E-128 | YP_002117783.1 |
| ORF4 | 1843 | 1947 | + | 34 | hypothetical protein BB757_005 | [Pseudomonas phage vB_Pae-TbilisiM32] | 98.20% | 2.00E-32 | AOZ64505.1 |
| ORF5 | 1950 | 2069 | + | 39 | hypothetical protein C5022_000008 | [Pseudomonas phage vB_PaeP_130_113] | 97.40% | 1.00E-17 | AVX47611.1 |
| ORF6 | 2148 | 2516 | + | 122 | hypothetical protein TM32_0004 | [Pseudomonas phage vB_Pae-TbilisiM32] | 99.10% | 1.00E-79 | YP_006299926.1 |
| ORF7 | 2503 | 2730 | + | 75 | hypothetical protein | [Pseudomonas phage vB_PaeP_PAO1_1-15pyo] | 98.80% | 2.00E-52 | CEF89886.1 |
| ORF8 | 2727 | 2912 | + | 61 | hypothetical protein | [Pseudomonas phage PAXYB1] | 98.40% | 5.00E-34 | ARB06187.1 |
| ORF9 | 2909 | 3088 | + | 59 | hypothetical protein | [Pseudomonas phage vB_PaeP_PAO1_1-15pyo] | 100% | 4.00E-35 | CEF89886.1 |
| ORF10 | 3088 | 3381 | + | 97 | hypothetical protein TM32_0007 | [Pseudomonas phage vB_Pae-TbilisiM32] | 96% | 9.00E-61 | YP_006299929.1 |
| ORF11 | 3378 | 3614 | + | 78 | hypothetical protein TM32_0008 | [Pseudomonas phage vB_Pae-TbilisiM32] | 100% | 1.00E-47 | YP_006299930.1 |
| ORF12 | 3632 | 3925 | + | 97 | hypothetical protein TM32_0009 | [Pseudomonas phage vB_Pae-TbilisiM32] | 99% | 2.00E-61 | YP_006299931.1 |
| ORF13 | 4004 | 4420 | + | 138 | hypothetical protein phiNFS_11 | [Pseudomonas phage phiNFS] | 100% | 1.00E-92 | AMQ66149.1 |
| ORF14 | 4489 | 4848 | + | 119 | hypothetical protein phiNFS_12 | [Pseudomonas phage phiNFS] | 100% | 3.00E-78 | AMQ66150.1 |
| ORF15 | 4851 | 5660 | + | 269 | putative DNA binding protein | [Pseudomonas phage phiKMV] | 100% | 0 | NP_877451.1 |
| ORF16 | 5930 | 6472 | + | 180 | hypothetical protein PT2_gp16 | [Pseudomonas phage PT2] | 100% | 9.00E-131 | YP_002117795.1 |
| ORF17 | 6462 | 6590 | + | 42 | hypothetical protein | [Pseudomonas phage LKD16] | 100% | 6.00E-21 | YP_001522803.1 |
| ORF18 | 6658 | 7482 | + | 274 | DNA primase | [Pseudomonas phage vB_Pae-TbilisiM32] | 99.64% | 0 | YP_006299937.1 |
| ORF19 | 7451 | 8719 | + | 422 | DNA helicase | [Pseudomonas phage vB_Pae-TbilisiM32] | 100% | 0 | YP_006299938.1 |
| ORF20 | 8709 | 9326 | + | 205 | hypothetical protein phiKMVp16 | [Pseudomonas phage phiKMV] | 100% | 3.00E-149 | NP_877455.1 |
| ORF21 | 9326 | 10273 | + | 315 | DNA ligase | [Pseudomonas phage phiKMV] | 100% | 0 | NP_877456.1 |
| ORF22 | 10270 | 10605 | + | 111 | hypothetical protein phiKMVp18 | [Pseudomonas phage phiKMV] | 100% | 5.00E-75 | NP_877457.1 |
| ORF23 | 10602 | 13025 | + | 807 | putative DNA polymerase | [Pseudomonas phage phiKMV] | 100% | 0 | NP_877458.1 |
| ORF24 | 13022 | 13333 | + | 103 | hypothetical protein phiKMVp20 | [Pseudomonas phage phiKMV] | 100% | 8.00E-69 | NP_877459.1 |
| ORF25 | 13390 | 14439 | + | 349 | hypothetical protein PT2_gp25 | [Pseudomonas phage PT2] | 100% | 0 | YP_002117804.1 |
| ORF26 | 14439 | 15380 | + | 313 | 5' to 3' exonuclease | [Pseudomonas phage vB_Pae-TbilisiM32] | 100% | 0 | YP_006299945.1 |
| ORF27 | 15370 | 15810 | + | 146 | putative DNA endonuclease VII | [Pseudomonas phage phikF77] | 100% | 4.00E-105 | YP_002727845.1 |
| ORF28 | 15807 | 16853 | + | 348 | hypothetical protein | [Pseudomonas phage vB_PaeP_PAO1_1-15pyo] | 100% | 0 | CEF89903.1 |
| ORF29 | 16863 | 17234 | + | 123 | hypothetical protein | [Pseudomonas phage vB_PaeP_PAO1_1-15pyo] | 100% | 9.00E-86 | CEF89904.1 |
| ORF30 | 17227 | 17577 | + | 116 | hypothetical protein | [Pseudomonas phage phikF77] | 98.28% | 2.00E-76 | YP_002727848.1 |
| ORF31 | 17586 | 20033 | + | 815 | DNA-dependent RNA polymerase | [Pseudomonas phage PT2] | 99.39% | 0 | YP_002117810.1 |
| ORF32 | 20194 | 20445 | + | 83 | hypothetical protein phiKMVp27 | [Pseudomonas phage phiKMV] | 100% | 1.00E-52 | NP_877466.1 |
| ORF33 | 20445 | 20918 | + | 157 | hypothetical protein phiKMVp28 | [Pseudomonas phage phiKMV] | 100% | 5.00E-114 | NP_877467.1 |
| ORF34 | 20884 | 21159 | + | 91 | putative structural protein | [Pseudomonas phage phiKMV] | 98.90% | 3.00E-55 | NP_877468.1 |
| ORF35 | 21171 | 22703 | + | 510 | head-tail connector protein | [Pseudomonas phage phiKMV] | 100% | 0 | NP_877469.1 |
| ORF36 | 22707 | 23675 | + | 322 | putative scaffolding protein | [Pseudomonas phage phiKMV] | 100% | 0 | NP_877470.1 |
| ORF37 | 23728 | 24735 | + | 335 | capsid protein | [Pseudomonas phage phiKMV] | 100% | 0 | NP_877471.1 |
| ORF38 | 24832 | 25386 | + | 184 | tail tubular protein A | [Pseudomonas phage phiKMV] | 100% | 5.00E-132 | NP_877472.1 |
| ORF39 | 25389 | 27869 | + | 826 | tail tubular protein B | [Pseudomonas phage phiKMV] | 100% | 0 | NP_877473.1 |
| ORF40 | 27869 | 28414 | + | 181 | putative internal virion protein A | [Pseudomonas phage phiKMV] | 100% | 1.00E-124 | NP_877474.1 |
| ORF41 | 28414 | 31110 | + | 898 | structural protein | [Pseudomonas phage phiKMV] | 99.89% | 0 | NP_877475.1 |
| ORF42 | 31114 | 35127 | + | 1337 | internal core protein | [Pseudomonas phage phiKMV] | 100% | 0 | NP_877476.1 |
| ORF43 | 35129 | 35884 | + | 251 | tail fiber protein | [Pseudomonas phage phiKMV] | 100% | 0 | NP_877477.1 |
| ORF44 | 35884 | 36324 | + | 146 | hypothetical protein phiKMVp39 | [Pseudomonas phage phiKMV] | 100% | 3.00E-101 | NP_877478.1 |
| ORF45 | 36314 | 37192 | + | 292 | hypothetical protein phiNFS_41 | [Pseudomonas phage phiNFS] | 99.66% | 0 | AMQ66179.1 |
| ORF46 | 37189 | 37779 | + | 196 | hypothetical protein phiNFS_42 | [Pseudomonas phage phiNFS] | 100% | 2.00E-143 | AMQ66180.1 |
| ORF47 | 37779 | 38084 | + | 101 | terminase small subunit | [Pseudomonas phage LUZ19] | 99% | 0 | YP_001671987.1 |
| ORF48 | 38094 | 39899 | + | 601 | terminase large subunit | [Pseudomonas phage LUZ19] | 98% | 0 | YP_001671988.1 |
| ORF49 | 39896 | 40096 | + | 66 | putative holin | [Pseudomonas phage phikF77] | 100% | 1.00E-38 | YP_002727867.1 |
| ORF50 | 40093 | 40575 | + | 160 | putative phage lysozyme | [Pseudomonas phage phiKMV] | 100% | 2.00E-115 | NP_877484.1 |
| ORF51 | 40533 | 40862 | + | 109 | Rz protein | [Pseudomonas phage phiKMV] | 100% | 5.00E-72 | NP_877485.1 |
| ORF52 | 40837 | 40950 | + | 37 | hypothetical protein | [Pseudomonas phage vB_PaeP_PAO1_Ab05] | 100% | 4.00E-18 | YP_009125750.1 |
| ORF53 | 40952 | 41266 | + | 104 | minor structural protein | [Pseudomonas phage phiKMV] | 100% | 9.00E-68 | NP_877486.1 |
| ORF54 | 41316 | 41510 | + | 64 | minor structural protein | [Pseudomonas phage phiKMV] | 100% | 8.00E-37 | NP_877487.1 |
