# Supplementary figures and images for: Characterization of a lytic Pseudomonas aeruginosa phage vB_PaeP_ASP23 and functional analysis of its lysin LysASP and holin HolASP
Source: Front Microbiol. 2023 Mar 15;14:1093668. doi: 10.3389/fmicb.2023.1093668 (PMC10045481; doi:10.3389/fmicb.2023.1093668)

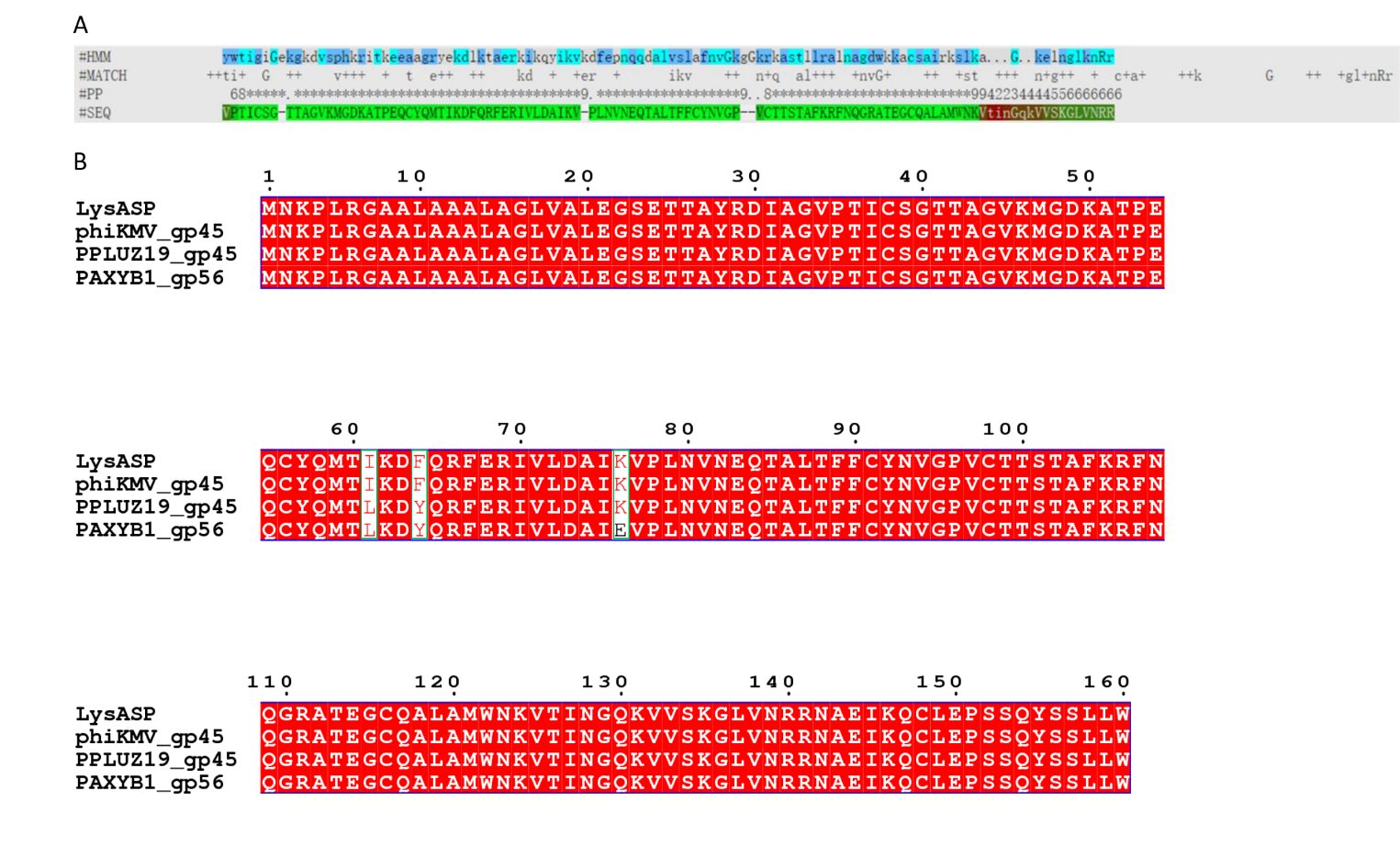

Supplement: Supplementary file 3 [file Image_1.TIF]

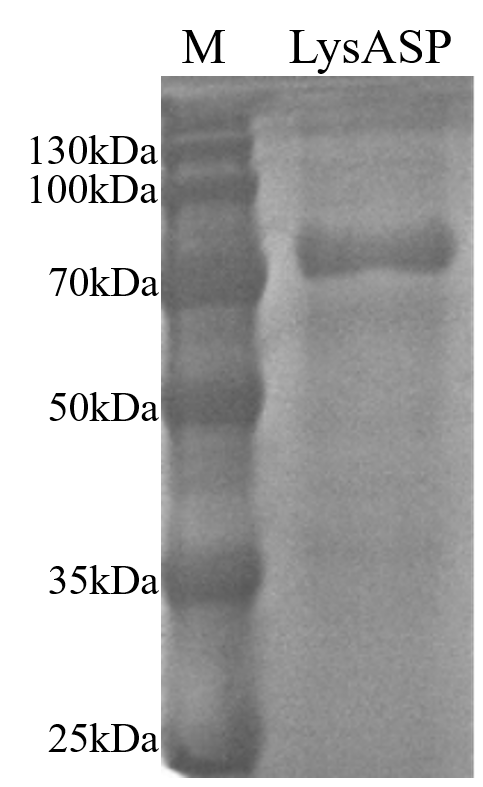

Supplement: Supplementary file 4 [file Image_2.TIF]

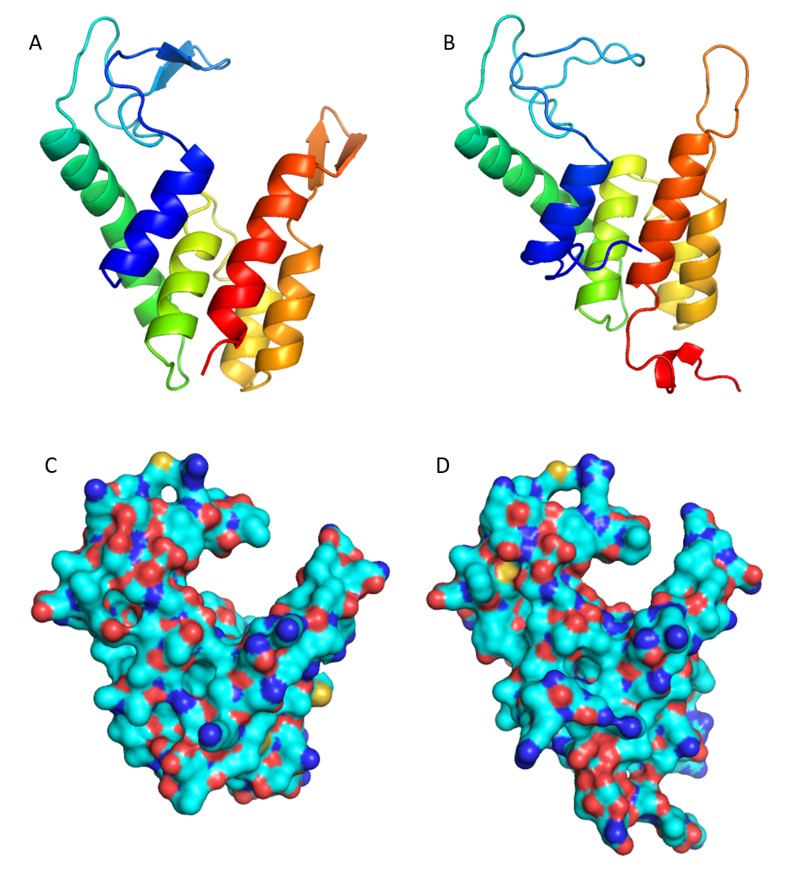

Supplement: Supplementary file 5 [file Image_3.JPEG]

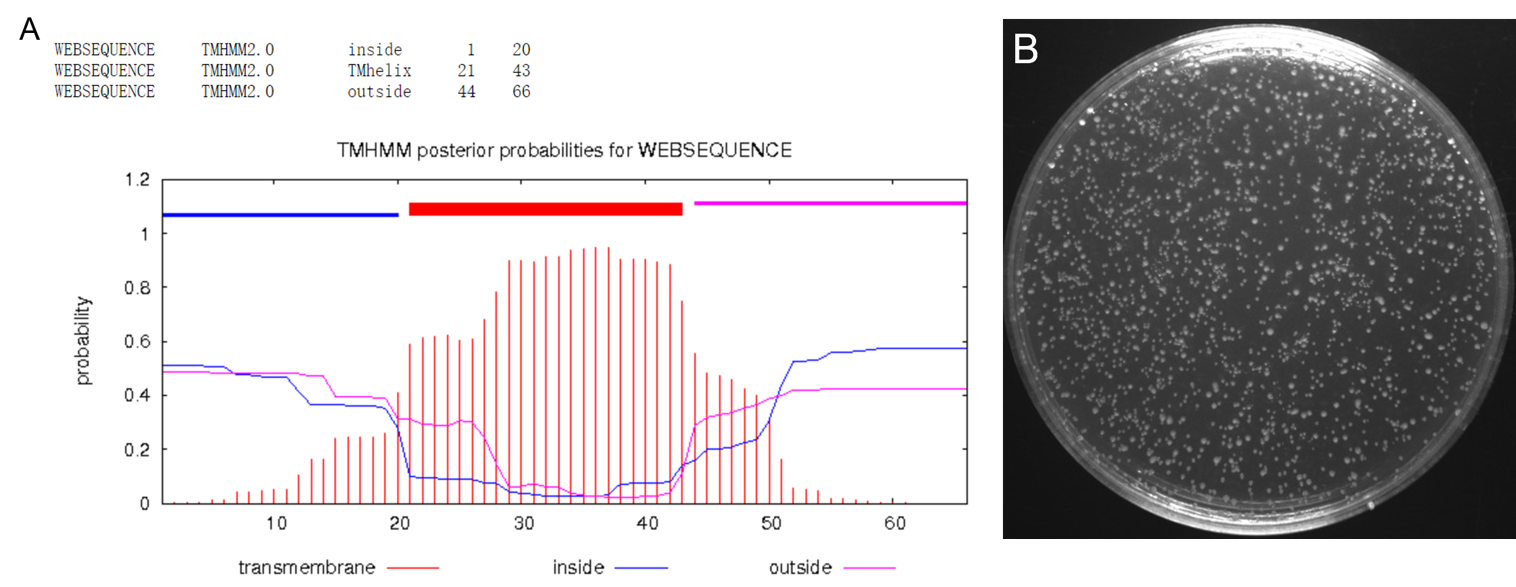

Supplement: Supplementary file 6 [file Image_4.PNG]
